# The timing of herbivore-induced volatile emission in black poplar (*Populus nigra*) and the influence of herbivore age and identity affect the value of individual volatiles as cues for herbivore enemies

**DOI:** 10.1186/s12870-014-0304-5

**Published:** 2014-11-28

**Authors:** Andrea Clavijo McCormick, G Andreas Boeckler, Tobias G Köllner, Jonathan Gershenzon, Sybille B Unsicker

**Affiliations:** Department of Biochemistry, Max Planck Institute for Chemical Ecology, Hans-Knöll-Straβe 8, 07745 Jena, Germany

**Keywords:** Diurnal rhythm, Herbivore-induced plant volatiles (HIPV), Herbivore feeding pattern, Lepidoptera, Salicaceae, Signaling molecules in indirect defense, Tree defense

## Abstract

**Background:**

The role of herbivore-induced plant volatiles as signals mediating the attraction of herbivore enemies is a well-known phenomenon. Studies with short-lived herbaceous plant species have shown that various biotic and abiotic factors can strongly affect the quantity, composition and timing of volatile emission dynamics. However, there is little knowledge on how these factors influence the volatile emission of long-lived woody perennials.

The aim of this study was to investigate the temporal dynamics of herbivore-induced volatile emission of black poplar (*Populus nigra*) through several day-night cycles following the onset of herbivory. We also determined the influence of different herbivore species, caterpillars of the gypsy moth (*Lymantria dispar*) and poplar hawkmoth (*Laothoe populi*), and different herbivore developmental stages on emission.

**Results:**

The emission dynamics of major groups of volatile compounds differed strikingly in response to the timing of herbivory and the day-night cycle. The emission of aldoximes, salicyl aldehyde, and to a lesser extent, green leaf volatiles began shortly after herbivore attack and ceased quickly after herbivore removal, irrespective of the day-night cycle. However, the emission of most terpenes showed a more delayed reaction to the start and end of herbivory, and emission was significantly greater during the day compared to the night. The identity of the caterpillar species caused only slight changes in emission, but variation in developmental stage had a strong impact on volatile emission with early instar *L. dispar* inducing more nitrogenous volatiles and terpenoids than late instar caterpillars of the same species.

**Conclusions:**

The results indicate that only a few of the many herbivore-induced black poplar volatiles are released in tight correlation with the timing of herbivory. These may represent the most reliable cues for herbivore enemies and, interestingly, have been shown in a recent study to be the best attractants for an herbivore enemy that parasitizes gypsy moth larvae feeding on black poplar.

**Electronic supplementary material:**

The online version of this article (doi:10.1186/s12870-014-0304-5) contains supplementary material, which is available to authorized users.

## Background

Herbivory induces dramatic changes in the volatile emission of plants. This phenomenon has been reported for many plant species from different orders, and possibly originated in photosynthetic bacteria long before the appearance of eukaryotic cells, leading to the belief that this is an ancestral feature of plants [1,2]. Herbivore-induced plant volatiles are well known to attract predators and parasitoids of herbivores and so have been frequently termed a “cry for help” from the plant to reduce herbivore pressure [2-4]. However, it is still unclear if herbivore enemy recruitment has a real fitness benefit for the plant or if plant volatiles are reliable cues for natural enemies of herbivores [5-7]. Major limitations in understanding the ecological roles of plant volatiles are the complexity of the emitted blends and our lack of knowledge on how insects perceive and process olfactory information [4].

One interesting aspect about volatile emission upon herbivory is its dynamic nature. Volatile emission patterns change during the course of herbivory with variation in how soon compounds are emitted after the start of herbivory [8-12], how soon emission decreases after herbivory stops [9,10,13] and changes in day and night cycles [14]. The emission patterns of abundant herbivore-induced volatiles, such as green leaf volatiles (GLVs) and terpenoids, are well described in the literature. However, much less is known about compounds emitted in lower amounts, such as aromatic compounds and amino acid derivatives (nitrogen and sulfur containing compounds) [2,4,15,16], although there is evidence that such minor compounds could have a high ecological value for both herbivores and their natural enemies [17-20].

Herbivore enemies have been shown to use differences in plant volatile emission to successfully discriminate between host plant species or cultivars [21-26] and between plants under different physiological stress conditions [27]. Herbivore parasitoids and predators can also obtain detailed information from volatile cues about the nature of the attacking herbivore species, and its developmental stage or parasitization status [14,28-30]. The presence of multiple herbivores adds another level of complexity to volatile emission causing increased attraction of herbivore enemies in some cases [31-35].

Understanding how herbivore enemies respond to volatiles emitted by different plant-herbivore combinations will increase our understanding about the ecological roles of specific compounds, but there are many gaps in our knowledge of what affects volatile emission in such circumstances. For example, how the spectrum of volatiles is altered by different herbivore species or different feeding stages is seldom taken into account (but see [36]). Additionally, most studies on herbivore enemy recruitment focus on volatiles present at just one time point after herbivory starts (but see [37]).

Despite the long history of research on plant volatiles, most research has concentrated on herbaceous species and relatively few studies have explored the emission of herbivore-induced volatiles from woody perennial species and their ecological roles (e.g. [18,38-43]).

Among woody plants, poplar has become a model organism because of its ecological and economic importance. In addition, since the completion of the genome of *Populus trichocarpa* [44], many genetic, genomic, biochemical and molecular tools are now available and a growing amount of information is accumulating that has opened the doors to studying many aspects of poplar biology, including direct and indirect defense [18,45].

In a previous study, we documented the enormous diversity of volatile compounds emitted by black poplar (*Populus nigra*) upon herbivore attack and established that the parasitoid *Glyptapanteles liparidis,* which preferentially parasitizes second instar gypsy moth (*L. dispar*) caterpillars on black poplar, is attracted to minor nitrogen-containing volatiles emitted by poplar locally at the sites of herbivory. Parasitoid wasps were also attracted to these minor volatiles and green leaf volatiles when compounds were presented individually under field conditions, indicating that these substances might be important cues for a broad range of natural enemies of herbivores feeding on poplar trees [18]. However, in this earlier study, we did not explore the reasons why these compounds might be preferred by parasitoids over other more abundant poplar volatiles such as terpenoids.

We hypothesize that compounds which are important cues for herbivore enemies should possess certain traits. They should A) indicate the actual presence of the herbivore (being rapidly emitted after the onset of herbivory with emission ceasing quickly after herbivore departure), B) be emitted independently of light and dark conditions at times when herbivore enemies are foraging, and C) provide information about the identity, age and abundance of the herbivore. The aim of this study was to investigate the temporal dynamics of herbivore-induced volatile emission of black poplar (*Populus nigra*) during and after herbivory, and to investigate the differences in volatile emission in response to different herbivore species, developmental stages of a herbivore and amount of feeding. These data should help establish which compounds could be most useful sources of information for herbivore enemies.

## Results

### Temporal dynamics of volatile emission in black poplar after gypsy moth herbivory

**Figure 1:**
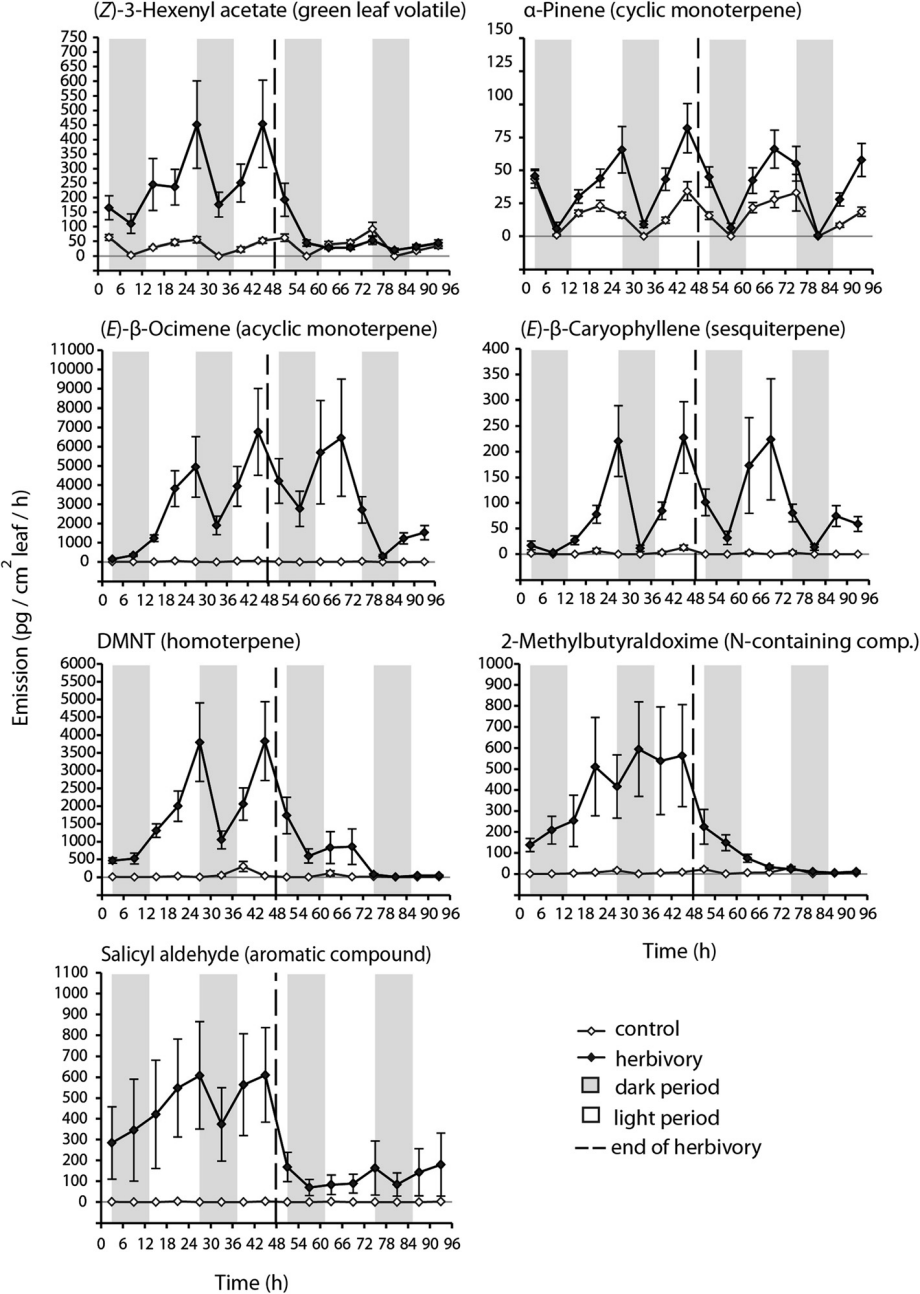
**Emission patterns representing the major chemical classes released by young trees upon herbivory by 4th instar larvae of** ***Lymantria dispar*** **(gypsy moth) or from undamaged controls over a 4-day experiment.** The graphs depict the rates of emission for individual compounds over the course of herbivory (initiated at the beginning of the experiment for herbivory treatment) as well as after herbivore removal. Volatiles were continuously sampled day and night in 6 h intervals. Means and ± SEM are given.

To investigate the diurnal patterns of black poplar (*Populus nigra*) volatile emission, we selected 20 compounds as representatives of each of the major classes of volatiles found in this species: green leaf volatiles (GLVs), monoterpenes (cyclic and acyclic), homoterpenes, sesquiterpenes, nitrogen-containing compounds and aromatic compounds. The volatile blend from undamaged trees was dominated by GLVs and cyclic monoterpenes, and these volatiles were almost exclusively emitted during light periods (Figure 1, Additional file 1: Figure S1). Feeding by 4^th^ instar larvae of the generalist herbivore *Lymantria dispar* caused an increased emission of all volatiles measured, although the extent of increase varied with the compound class, diurnal cycle, and the timing of herbivory.

GLVs such as (*Z*)-3-hexenyl acetate were emitted rapidly upon the onset of herbivory, and emission declined after herbivore removal. They were released both day and night, with a greater emission during the day. The emission of terpenoids was also greater during the day than at night, but the increase in emission after herbivory did not coincide with the onset of herbivory, but occurred only several hours after caterpillar damage had begun. Furthermore, terpenoids continued to be emitted several hours after herbivore removal and in some cases even until the end of the experiment 48 hours later (Figure 1, Additional file 1: Figure S1). Among the terpenes, cyclic monoterpenes showed only a modest increase in emission after induction (roughly two-fold). By contrast, acyclic monoterpenes, sesquiterpenes, and the homoterpene DMNT, which were only present in minute amounts in the headspace of uninfested plants, showed a many-fold increase (e.g. 7000-fold for (*E*)-β-ocimene, 4000-fold for DMNT and 250-fold for (*E*)-β-caryophyllene) after herbivory (Figure 1, Additional file 1: Figure S1).

Of the nitrogen-containing compounds, the emission of 2-methylbutyraldoxime was induced immediately by herbivory and increased to its highest levels during the first full light period. The rate of emission was not influenced by the light or dark period, and it declined to baseline levels after herbivory ended (Figure 1). The emission patterns of two other nitrogen-containing compounds, benzyl cyanide and indole, were different in displaying significant diurnal rhythms (emission 2-3-fold greater during the day as during the night) and a less rapid decline after caterpillars were removed (Additional file 1: Figure S1).

Among the aromatic compounds, salicyl aldehyde was emitted almost from the onset of herbivory in substantial rates, both day and night, ceasing abruptly after herbivore removal (Figure 1). Two other aromatic compounds, benzyl alcohol and benzene ethanol, showed much more of a biphasic emission pattern, elevated during the day and reduced at night (Additional file 1: Figure S1). Emission was induced by herbivory more slowly than for salicyl aldehyde and stayed at significantly higher emission levels than in controls until almost the end of the experiment rather than declining rapidly after herbivory stopped as for salicyl aldehyde.

### Effect of herbivore species, its developmental stage, and feeding intensity on volatile emission

**Figure 2:**
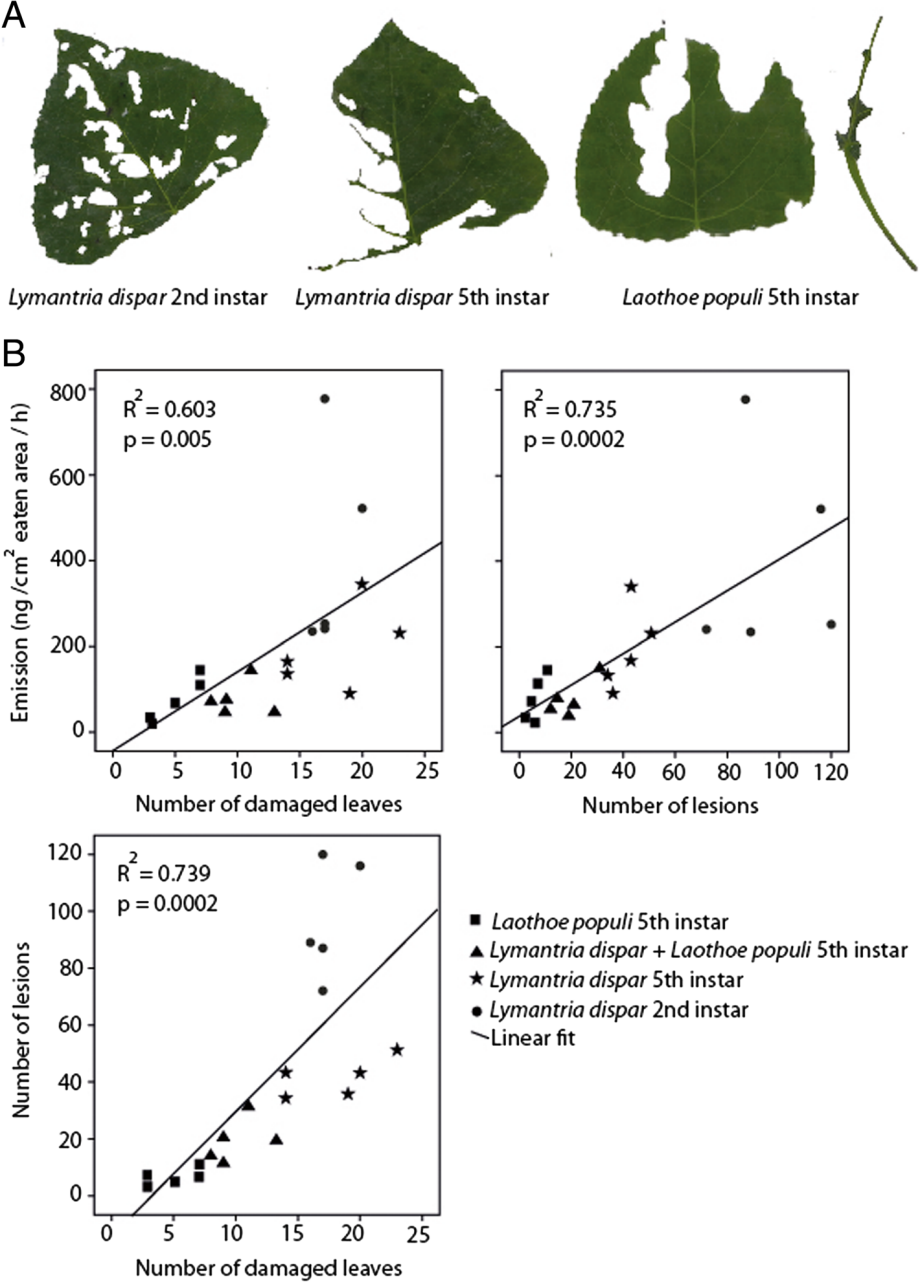
**Differences in insect feeding patterns and effect on volatile emission. A**. Pictures of the characteristic feeding damage caused by second instar *Lymantria dispar*, fifth instar *L. dispar* and fifth instar *Laothoe populi* on *Populus nigra* leaves. **B**. Correlation between two insect feeding parameters (number of leaves damaged and number of lesions) and total volatile emission of *P. nigra* leaves (combined emission of the 20 compounds investigated) in relation to the amount of leaf area eaten. Correlation between the two feeding parameters is also shown. Herbivory treatments are depicted by different symbols.

In comparing the herbivory of 5^th^ instar *L. dispar* larvae to that of 2^nd^ instar *L. dispar* and larvae of another lepidopteran, the specialist *Laothoe populi*, we observed that the three treatments had very characteristic damage patterns (Figure 2A). For example, 5^th^ instar *L. populi* larvae caused few, but very extensive lesions in a few leaves, often completely consuming the whole leaf blade. Fifth instar *L. dispar* caterpillars also caused extensive lesions on a few leaves, but mostly avoided the leaf venation and only rarely consumed whole leaf blades (Figure 2A). Furthermore 5^th^ instar *L. dispar* caterpillars moved more often from one leaf to another thus damaging more leaves overall than *L. populi*. In contrast, second instar *L. dispar* caterpillars caused numerous small lesions and frequently changed feeding position causing minor to moderate damage on a larger number of leaves. When fifth instar *L. dispar* and *L. populi* were combined, there was an intermediate damage pattern between that of both herbivores measured separately (Figure 2B).

To quantify the feeding differences among herbivore treatments in relation to volatile emission, we calculated the number of damaged leaves and number of lesions as observed in each treatment. We found a significant positive correlation between total volatile emission and the two parameters: number of damaged leaves and number of lesions (R^2^ = 0.603, p = 0.005 and R^2^ = 0.735, p = 0.0002, respectively), as well as a significant correlation between these two damage parameters (R^2^ = 0.739, p = 0.0002) (Figure 2B). Therefore we used principal component analysis as a factor reduction technique to combine these two parameters into a single component which we termed feeding intensity. Then we applied a generalized least square model (GLS) to calculate the effect of the herbivory treatment (larval species and instar), the feeding intensity (regardless of treatment), and their interaction on the rate of emission of each of the 20 studied compounds (Table 1). Emission was calculated relative to total leaf area consumed in each treatment to control for variation in the extent of herbivory among treatments.

**Table 1:** **Effect of herbivore identity, feeding intensity and their interaction on black poplar volatile emission**

**Compound**		**Interaction (feeding intensity x Herbivore treatment)**		**Herbivory treatment**		**Feeding intensity**	
	**Variance structure**	**Likelihood ratio**	**p. value**	**Likelihood ratio**	**p. value**	**Likelihood ratio**	**p. value**
*Monoterpenes*							
α-Pinene (cyclic)	7	0.906	0.824	15.227	0.002**	15.393	<0.001***
Camphene (cyclic)	2	0.999	0.802	8.246	0.038*	8.883	0.003**
Myrcene (cyclic)	4	0.642	0.887	8.364	0.04*	7.533	0.006**
Borneol (cyclic)	2	0.779	0.855	6.925	0.074	9.052	0.003**
(*Z*)-Ocimene (acyclic)	8	3.951	0.267	7.286	0.063	8.424	0.004**
(*E*)-β-Ocimene (acyclic)	4	1.384	0.709	9.797	0.020*	14.036	<0.001***
Linalool (acyclic)	2	1.441	0.696	2.456	0.483	11.012	<0.001***
*Homoterpene*							
DMNT	8	7.797	0.051	3.786	0.286	0.444	0.505
*Sesquiterpenes*							
(*E*)-β-Caryophyllene	2	0.667	0.881	11.371	0.01*	4.334	0.037*
α-Humulene	2	1.526	0.676	9.014	0.029*	1.676	0.196
Nerolidol	4	6.410	0.093	10.387	0.016*	12.891	<0.001***
*Green leaf volatiles*							
(*Z*)-3-Hexenyl acetate	2	0.656	0.884	6.067	0.108	6.454	0.011*
(*Z*)-3-Hexenol	1	2.284	0.516	16.015	0.001**	0.138	0.71
*N-containing comp.*							
2-Methylbutyraldoxime	7	0.522	0.914	10.821	0.013*	4.454	0.035*
3-Methylbutyraldoxime	4	0.536	0.911	14.950	0.002**	10.335	0.001**
Benzyl cyanide	3	2.723	0.466	10.852	0.013*	9.464	0.002**
Indol	3	2.136	0.545	9.688	0.021*	1.537	0.215
*Aromatic compounds*							
Salicyl aldehyde	8	8.734	0.033*	13.706	0.003**	0.535	0.464
Benzyl alcohol	4	4.867	0.182	4.624	0.202	6.770	0.386
Benzene ethanol	4	8.003	0.046*	7.629	0.054	0.703	0.402

For each parameter the F and p values are given. Asterisks indicate significant differences, p < 0.001 = ***, p < 0.01 = **, p < 0.05 = *, no asterisk = not significant. Compounds are grouped according to their chemical classes, the second column shows the variance structure with the lowest Akaike Information Criterion (AIC), which was used in the Generalized Least Square model (GLS). Variance structures tested were as follows: 1. *varFixed* variance for feeding intensity, 2. *varIdent* variance for herbivory treatment, 3. *varPower* variance for herbivory treatment, 4. *varExp* variance for feeding intensity, 5. *varConstPower* for feeding intensity, 6. *varConstPower* for feeding intensity and herbivory treatment, 7. Combined variance (*varIdent* for herbivory treatment, *varFixed* for feeding intensity) and 8. Combined variance (*varIdent* for *herbivory treatment*, *varExp* for feeding intensity). A detailed description of the variance structures is given by [80].

**Figure 3:**
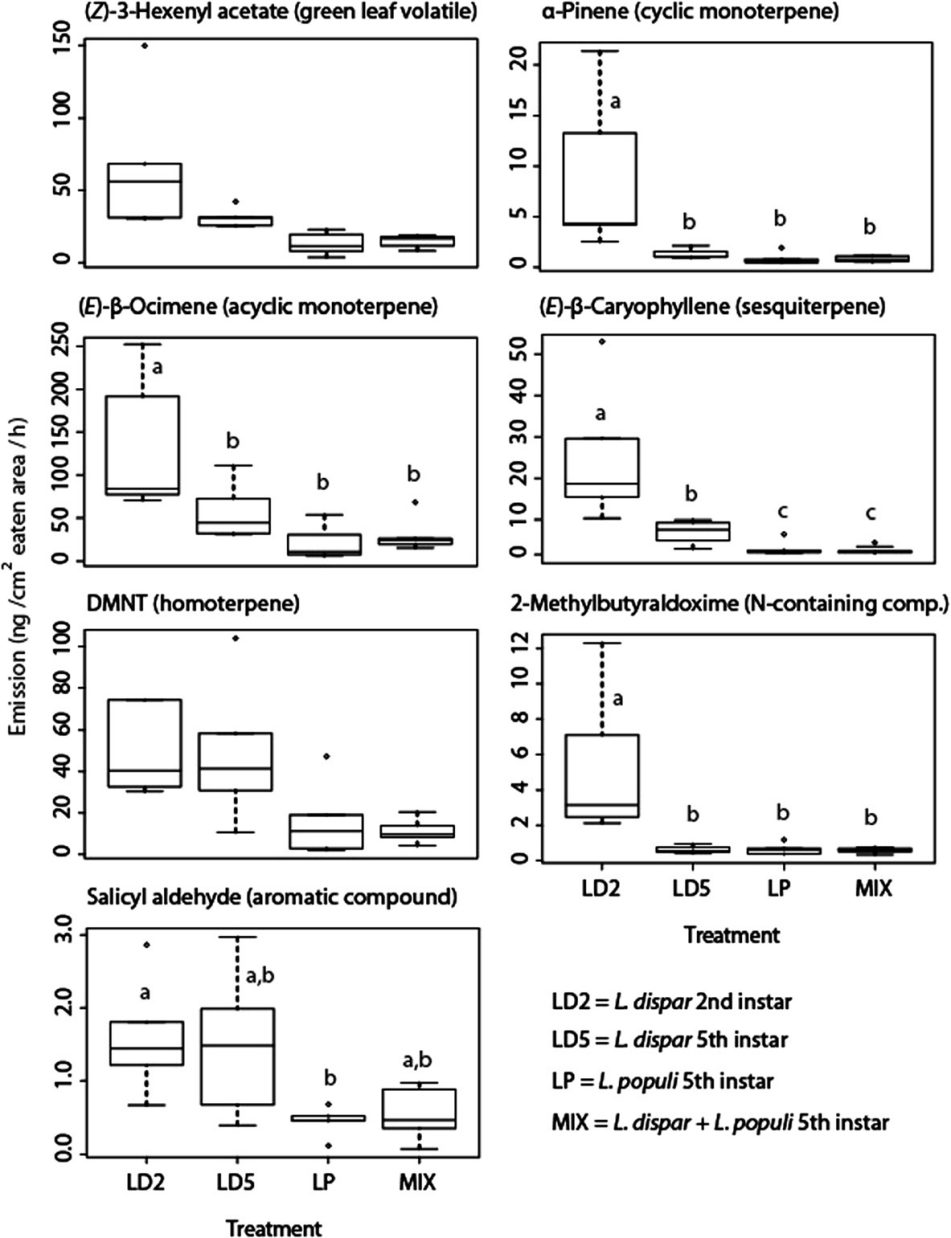
**Effect of herbivore identity and developmental stage on volatile emission of** ***Populua nigra*** **.** Four treatments include *Lymantria dispar* (2nd instar), *L. dispar* (5th instar), *Laothoe populi* (5th instar), and a mixture of *L. dispar* (5th instar) and *L. populi* (5th instar). Box-plots showing the same letter are not statistically significant from one another after a Tukey test performed on the fitted values after applying a GLS model, excluding the effect of the feeding intensity. P values are given in Table 1. Plots showing no letters indicate that there was no effect of the treatment on volatile emission.

In comparing *P. nigra* volatiles among treatments, only four compounds differed significantly in emission upon feeding by the two caterpillar species tested (the specialist *L. populi* and the generalist *L. dispar* both 5^th^ instar): (*E*)-β-caryophyllene, 3-methylbutyraldoxime, myrcene and nerolidol (Figure 3), all emitted in greater abundance after damage by *L. dispar*. Four compounds were also different between combined damage by the two herbivore species vs. damage by the generalist herbivore alone: (*E*)-β-caryophyllene, 3-methylbutyraldoxime, (*Z*)-3-hexenol and nerolidol (Figure 3). These compounds were emitted in higher amounts by *L. dispar* than by the two species combined. The emission in the combined damage treatment did not differ significantly from that induced by the specialist herbivore (*L. populi*) alone (Figure 3, Additional file 2: Figure S2). Herbivore instar had very strong effect on volatile emission caused by *L. dispar*: early instar *L. dispar* induced significantly more emission of nitrogen-containing volatiles and most terpenoids than late instar *L. dispar* and *L. populi* (Figure 3, Additional file 2: Figure S2).

**Figure 4:**
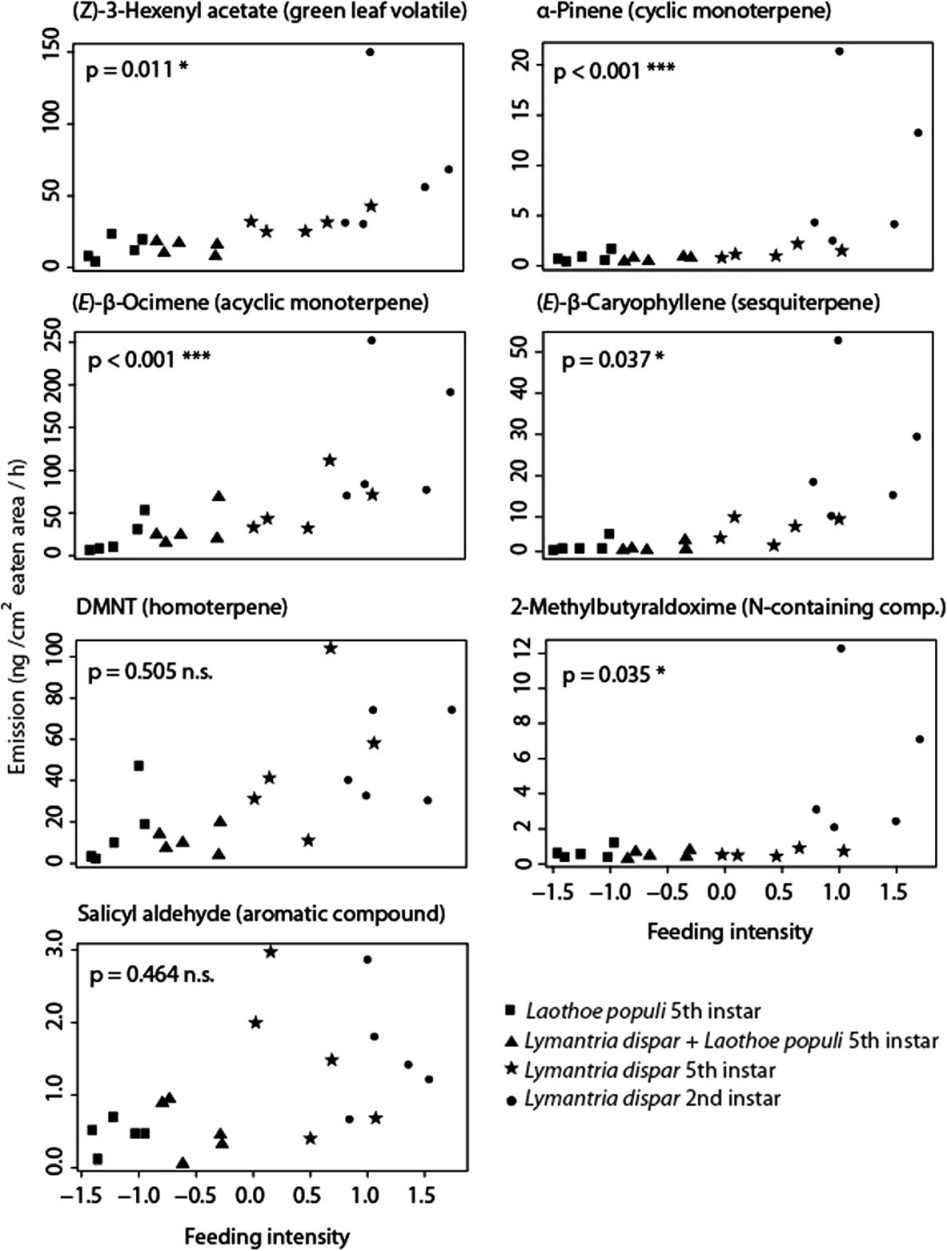
**Effect of feeding intensity during various herbivory treatments on volatile emission of** ***Populus nigra*** **compounds.** P values indicate significant differences after applying a GLS model (excluding the effect of herbivore identity). p < 0.001 = ***, p < 0.05 = *, n.s. = not significant. Herbivory treatments are depicted by different symbols.

The feeding intensity had also a significant direct effect on the emission of the majority of black poplar volatiles tested: all monoterpenes, the sesquiterpenes nerolidol and (*E*)-β-caryophyllene, all nitrogen containing volatiles excluding indole and the GLV (*Z*)-3-hexenyl acetate (Figure 4, Additional file 3: Figure S3, Table 1). Interestingly the emission of DMNT, which is one of the most abundant herbivore induced volatiles, was shown not to be influenced by feeding intensity or the identity and developmental stage of the herbivore, which is also the case for the aromatic compounds benzyl alcohol and benzene ethanol (Table 1). For the interaction between herbivory treatment (herbivore identity and developmental stage) and feeding intensity, we only observed a significant effect for two aromatic compounds, salicyl aldehyde and benzene ethanol.

## Discussion and conclusions

### Major groups of herbivore-induced volatiles in poplar show different temporal emission patterns

The value of herbivore-induced plant volatiles as cues for herbivore enemies depends on how closely their emission correlates with the presence of herbivores. While some compounds were emitted almost immediately after the onset of herbivory and ceased emission soon after herbivory had stopped, others, especially terpenes, were first emitted only 12 hours after the beginning of herbivory and continued being emitted for a day or more after herbivory had stopped. These differences suggest very divergent mechanisms triggering and controlling the biosynthesis of these compounds [2,15,16]. There are also differences for the same compound class among different plant species. For instance, GLV emission is often considered to be restricted to the time when actual leaf damage occurs [46], but here (*Z*)-3-hexenol emission continued for 24 hours after herbivory had stopped (Additional file 1: Figure S1). The volatiles that are the most diagnostic cues for herbivore enemies, should be emitted as long as herbivores are present.

Variation of emission with day-night rhythm may also affect the value of volatiles as herbivore enemy attractants. The emission of most herbivore-induced and constitutive volatiles was found to vary strongly in a diurnal fashion. The terpenoids followed this trend especially well with emission being much higher in light vs. dark periods for all compounds measured. Previous work with herbaceous plants also found the emission of monoterpenes (C_10_), sesquiterpenes (C_15_) and homoterpenes (the C_15_-derived homoterpene DMNT) to be much higher in the day than the night [9,47]. A correlation with light may arise because much of the substrate for the biosynthesis of volatile terpenes arises from the methylerythritol phosphate pathway [48-50], which is closely connected with photosynthesis [51].

Other groups of compounds showed less clear trends in day-night emission patterns. Certain green leaf volatiles (GLVs) [(Z)-3-hexenyl acetate], nitrogen-containing compounds (benzyl cyanide, indole) and aromatic compounds (benzene ethanol) displayed a strong diurnal rhythm with more emission in the light, but other members of these groups showed weaker rhythms or none at all. GLVs are sometimes reported to be emitted independently of any diurnal rhythm [38,52] or only at night [47]. Diurnal variation in volatile emission has been reported for many compounds in a range of plant species, both herbaceous and woody, induced by herbivores or pathogens [9,47,53-56], including poplar [38,42], but the regulatory mechanisms are not known.

The significance of day-night variation for herbivore enemy attraction depends on the activity rhythms of enemies. If enemies are active throughout the 24 hr cycle, an emission pattern independent of light and dark, such as that of 2-methylbutyraldoxime, salicyl aldehyde or some GLVs, may be most advantageous. For enemies that are only active at specific periods, emission during those times is most critical.

### Emission varies in response to herbivore developmental stage, but not to herbivore species

In our study we found very few differences in volatile emission among black poplar fed upon by two different herbivore species, *Lymantria dispar* and *Laothoe populi*. Possible explanations for this lack of species-specificity are that the two lepidopteran species tested feed in the same manner and share similar elicitors in their saliva. In previous studies, feeding by leaf-chewing lepidopteran larvae and grasshoppers has induced similar blends of volatiles [12,57,58] suggesting that these volatiles are a general response to attack by chewing insects. In support of this suggestion is the fact that, despite the great diversity of herbivores, only a few elicitors triggering defense responses in plants have been identified in herbivore oral secretions so far [59-62].

In contrast to arthropod herbivores from a single feeding guild, such as leaf chewers or phloem feeders, it is likely that arthropods from different feeding guilds induce different patterns of plant volatile emission [63,64], although there are exceptions in the literature that challenge this idea [65,66]. If there are differences in feeding mode between younger and older larvae of a single species, these might also lead to differences in emission. In our study, we found that “feeding intensity” (a factor combining number of damaged leaves and number of lesions) differed between early and late instar *Lymantria dispar*. In fact, there was more similarity in feeding intensity between late instar *L. dispar* and late instar *Laothoe populi* than between early and late instar *L. dispar* which led to corresponding differences in volatile emission. Nitrogen-containing volatiles and most terpenes were emitted at greater rates from early vs. late instar *L. dispar*. Thus volatile emission profiles were more influenced by instar and damage intensity than the identity of the herbivore species. Alterations in emission profiles induced by feeding of different instars of a single herbivore have also been reported in previous studies [30,67,68], and may aid herbivore enemies in finding their favored prey or host stage. The differences between instars in our study may also be due to the fact that, although we controlled for herbivore weight, the number of feeding caterpillars was much higher in the second instar herbivory treatment than in the 5^th^ instar treatments. However, under natural conditions, many moth and butterfly caterpillars are gregarious early in development, and become solitary in late instars [69]. Thus the differences in our treatments reflect natural conditions.

When young black poplar trees were simultaneously attacked by late instar *L. dispar* and *L. populi* caterpillars the emission of a few compounds decreased in comparison to trees infested by late instar *L. dispar* alone. Previous studies have already documented attenuation in volatile emission upon multiple herbivore species attack, however, examples for enhanced volatile emission in response to simultaneous feeding by different species also exist [70].

Further studies on the effects of larval stage and simultaneous attack by different herbivore species on volatile emission are necessary to better define these differences and survey their impact on herbivore enemies.

### The potential role of black poplar volatiles in attraction of herbivore enemies

Our initial hypothesis was that plant volatile compounds employed as cues by herbivore enemies should: A) reliably indicate the actual presence of herbivores, B) be emitted independently of light or dark cycles as long as herbivore enemies are active, and C) provide specific information about the identity, developmental stage and abundance of the herbivore. Although no individual compound released from *P. nigra* meets all the requirements, 2-methylbutyraldoxime and salicyl aldehyde fulfill the first two requirements best, whereas a number of compounds are informative regarding herbivore identity (3-methylbutyraldoxime, myrcene, (*E*)-β-caryophyllene and nerolidol), herbivore instar (aldoximes, most terpenes) and herbivore abundance (most volatiles).

The list of volatiles that best meet the criteria to serve as good signals for enemies of *P. nigra* herbivores shows a remarkable correspondence with those compounds found previously to be attractive to the braconid koinobiont parasitoid *Glyptapanteles liparidis*, which is a specialist on early instar *L. dispar* caterpillars. The aldoximes, 2- and 3-methylbutyraldoxime, were the only compounds showing attraction in laboratory bioassays, while 2-methylbutyraldoxime (3-methylbutyraldoxime was not tested), benzyl cyanide, (*Z*)-3-hexenol, (*Z*)-3-hexenyl acetate and linalool were attractive to a community of different parasitoid species in a natural *P. nigra* stand [18]. It would be interesting to know the major attractive cues for other enemies of *L. dispar*. The importance of individual herbivore-induced volatiles to herbivore enemies may also depend on their degree of host or prey specificity [71]. Generalist parasitoids and predators might orient towards abundant widespread compounds which generally signal herbivory (such as GLVs), whereas specialists may benefit from responding only to more specific compounds (such as aldoximes). Even though the differences in black poplar volatile emission upon damage by late instar *L. populi* and *L. dispar* are minor, parasitoids may still be able to locate their prey under natural conditions, as they possess very sensitive olfactory systems to detect slight changes in volatile cues that we cannot detect with our analytical devices.

In the case of koinobiont parasitoids which develop inside a living host, there is a preference to oviposit in early instar larvae to prevent the risk of encapsulation as well as to allow the completion of the endoparasitic larval stage which would not be possible if the host entered pupation [72,73]. In this sense, compounds signaling early instar damage should be of great importance for koinobiont parasitoids.

The emission patterns of herbivore-induced volatiles may also reflect other roles of these substances in the plant. Plant volatiles have been implicated in direct defense against herbivores [74], communication within and among plants [75], and resistance to abiotic stresses, such as high light and temperature [76]. The importance of some of these roles could vary during the diurnal cycle. For example, since light and high temperature stresses would occur during the day, volatiles such as isoprene and monoterpenes involved in resistance to these stresses might be emitted in greater amounts during the day.

### Critical conclusion

Upon herbivore damage, plants typically emit a large, diverse blend of volatile compounds that have been shown to have importance in direct defense against herbivores and the attraction of herbivore enemies. In black poplar, a few individual compounds of the blend have been shown to be active in enemy attraction [18]. Here we show that these active compounds may have been selected as cues by herbivore enemies because they are more reliable indicators of herbivore presence and provide information about the age and identity of the damaging species.

## Methods

### Plants & insects

#### Populus nigra

Black poplar trees were grown from stem cuttings obtained from old-growth trees and raised under summer conditions in a climate chamber (~14:10 h day:night photoperiod, 22°C day - 19°C night, 60% humidity). The light period started at 6:00 a.m. and ended at 8:00 p.m. Cuttings were planted in 2 L pots containing a 2:2:1 mixture of clay, humus and sand. Fertilizer and water were applied regularly until the experiment started.

#### Lymantria dispar

*L. dispar* caterpillars were hatched from egg clutches (kindly provided by Melody Keena and Hanna Nadel from the, US Department of Agriculture - Mill Pond Road Hamden, CT and Buzzard’s Bay, MA) and reared on artificial gypsy moth diet (MP Biomedicals LLC, Illkirch, France) until two days before the experiments started, where they were then fed with *P. nigra* leaves to get adapted to this food source. All caterpillars were maintained in a climate chamber with the same photoperiod, temperature and relative humidity conditions as described above.

#### Laothoe populi

*L. populi* caterpillars were hatched from eggs (purchased from the Lepidoptera Breeders Association, Seaford, UK) and reared on fresh poplar leaves at ambient temperatures in the laboratory.

### Volatile collection and analysis

#### Temporal dynamics experiment

To investigate the diurnal variation of volatile emission in poplar, volatiles were collected in a climate chamber using a push-pull system that consisted of a circular Plexiglas top (Ø 26 cm) attached to a cylindrical PET bag (Ø 26 cm, 50 cm height). Two holes were drilled through the top to hold the valves for incoming and outgoing air. A young tree (~40 cm tall, 2 months old) was introduced into the system through the bottom opening and the PET-bag was fastened to the pot with a cable binder. During the volatile collection, charcoal purified air was pumped through Teflon tubing into the system at a flow rate of 2.5 L min^−1^. At the same time, 1.5 L min^−1^ of air from the plant headspace was pumped out of the system through a Teflon tube passing through a 20 mg Super-Q (Alltech, FL, USA) filter to absorb volatiles. The abiotic conditions in the climate chamber were kept the same as described above. Ten trees were assigned to each of two treatments (herbivory, control) and placed inside the collection system. Fifteen 4^th^ instar gypsy moth caterpillars were released on the trees in the herbivore treatment shortly before the first volatile collection. The first volatile collection started at 5 pm with 3 h light period remaining. Volatile emission was continuously sampled in 6 h intervals for a total of 96 h, both during day and night. Gypsy moth caterpillars were removed from herbivore-treated trees after 48 h. By the end of the experiment, all leaves were excised and photographed to determine the leaf area as described in [77]. Volatiles were eluted from Super-Q Traps with 200 μL dichloromethane containing 10 ng/μL of nonyl acetate as an internal standard. A portion (2 μL) of the eluate was splitlessly injected in a GC/MS equipped with a 30 m × 250 μm × 0.25 μm DB5-MS column (Wicom GmbH, Heppenheim, Germany). The injector was held at 230°C and helium was used as a carrier gas at 1 mL/min. The oven temperature of the GC/MS was held at 50°C for 3 minutes after injection and then increased to 95°C at a rate of 4°C/min. Afterwards, the oven was heated to 145°C with a 15°C/min gradient and then to 180°C with a 10°C/min gradient. Finally, the oven temperature was held for 3 min at 300°C. Mass spectra were recorded with a 3 min solvent delay using a Hewlett-Packard MSD 5973 mass spectrometer (transfer line temp: 230°C, source temp: 230°C, quadrupole temp: 150°C, ionization energy: 70 eV, mass range: 40–500 m/z). Compounds were identified by comparing their retention time to those of authentic standards. Quantification was carried out by mass spectrometry since the emission of some volatiles during dark periods turned out to be too low for flame ionization detection. This however, limited the quantification to compounds that could be acquired commercially in acceptable purity (>90%). Selected ion monitoring was used for quantification in a way that a specific m/z of each compound was referenced to the m/z = 69 of the internal standard. The compound and m/z specific response factors required for absolute quantification were calculated from dilutions of the authentic standards in dichloromethane with a constant internal standard concentration of 8.64 ng/μL. For each compound, two response factors were averaged from two six point calibration curves, one for a lower concentration range (0.2-1 ng/μL) and one for a higher concentration range (1–10 ng/μL). The amount of volatiles emitted was normalized to the leaf area.

#### Effect of herbivore species and developmental stage experiment

To investigate the differences in volatile emission of black polar trees infested with different species of caterpillars and different instars of the same species, five trees were assigned to each of the following treatments: control (undamaged trees), *L. dispar* second instar herbivory (3000 mg of larval weight -approximately 60 caterpillars, LD2), *L. dispar* fifth instar (3000 mg of larval weight – 3 to 4 caterpillars, LD5), *L. populi* fifth instar (3000 mg of larval weight – 3 to 4 caterpillars, LP), mixed herbivory (3000 mg of larval weight 1500 mg for *L. populi* and 1500 for *L. dispar –* 2 caterpillars of each species, MIX). Caterpillars were weighed, separated by groups and starved the day before the experiment. The experiment was conducted in a climate chamber under the same conditions as described above. At the beginning of the experiment, at 9:00 am the caterpillars were placed on the trees according to treatment. Volatiles were collected during four hours between 48 and 52 h after the herbivores were added. The caterpillars remained on the trees during volatile collection. The experimental setup for volatile collection and filter elution are described above. Qualitative and quantitative volatile analysis was conducted using an Agilent 6890 Series gas chromatograph coupled to an Agilent 5973 quadrupole mass selective detector (interface temp, 270°C; quadrupole temp, 150°C; source temp, 230°C; electron energy, 70 eV) and a flame ionization detector (FID) operated at 300°C, respectively. The constituents of the volatile bouquet were separated using a ZB-WAX column (Phenomenex, Aschaffenburg, Germany, 60 m × 0.25 mm × 0.15 μm) and He (MS) or H_2_ (FID) as carrier gas. A portion (1 μl) of the sample was injected without split at an initial oven temperature of 40°C. The temperature was held for 2 minutes and then increased to 225°C with a gradient of 5°C/min, held for another 2 minutes and then further increased to 250°C with 100°C/min and a hold for 1 min. Compounds were identified by comparison of retention times and mass spectra to those of authentic standards. The absolute amount of all compounds was determined based on their FID peak area in relation to the area of the internal standard using the effective carbon number (ECN) concept as described by Scanion and Willis [78]. We restricted our analyses to 20 compounds for which standards were available in high purity (>90%) (Table 1). After termination of the experiment, volatile collections of the caterpillars removed from the leaves along with the frass produced throughout the experiment were performed as described above (Additional file 4: Table S1). Leaves from individual trees were harvested separately, and photographed to determine the area of leaf damage as described in [77]. In addition we recorded the number of lesions and damaged leaves per tree.

### Statistical analyses

All statistical assumptions such as normal distribution and heteroscedasticity were checked. Throughout the manuscript means are always given with standard errors (SE). To determine the importance of volatiles emitted from *P. nigra* in characterizing the different herbivory treatments (*L. dispar* second instar, *L. dispar* fifth instar, *L. populi* fifth instar, and mixed herbivory), we combined the effect of the covariates “number of damaged leaves” and “number of lesions” by performing a principal component analysis for factor reduction as described in [79]. We termed the new variable feeding intensity. Due to the high variability among treatments and the fact that compounds showed different emission patterns, we tested eight models with different variance structures for each compound according to [80]. Model comparison was performed by a maximum likelihood ratio test using the Akaike Information Criterion (AIC) as a measure for the predictive power of the respective statistical model. The model with the lowest AIC value was then selected for the analysis. Table 1 gives an overview of the statistical models applied. For the selected model we applied a generalized least square model (GLS) to calculate effect of the herbivory treatment (different species, instars and combined damage), the feeding amount and their interaction on the emission of a given compound. Whenever the herbivory treatment was significantly different, we performed a Tukey test for comparison of means on the fitted values. Statistical analyses were performed using R 2.15.2 (R Development Core Team; http://www.r-project.org).

## Additional files

Additional file 1: Figure S1. Volatile emission pattern of thirteen further volatiles of *Populus nigra* foliage representing the major chemical classes released by young trees upon herbivory by fourth instar larvae of *Lymantria dispar* (gypsy moth) or from undamaged controls over a 4-day experiment. The graphs depict the rates of emission for individual compounds over the course of herbivory (initiated at the beginning of the experiment for herbivory treatment as well as after herbivore removal) during day and night in 6 h intervals. Means + SEM are given at the end of each measuring period. Additional file 2: Figure S2. Effect of herbivore identity and developmental stage on volatile emission of *Populus nigra* (for thirteen further volatile compounds), Four treatments include *Lymantria dispar* (2nd instar), *L, dispar* (5th instar), *Laothoe populi* (5th instar), and a mixture of *L, dispar* (5th instar) and *L, populi* (5th instar), Box-plots showing the same letter are not statistically significant from one another after a Tukey test performed on the fitted values after applying a GLS model, excluding the effect of the feeding intensity, P values are given in Table 1, Plots showing no letters indicate that there was no effect of the treatment on volatile emission. Additional file 3: Figure S3. Effect of feeding intensity during various herbivory treatments on volatile emission of Populus nigra compounds (for thirteen further volatile compounds), P values indicate significant differences after applying a GLS model (excluding the effect of herbivore identity), p < 0,001 = ***, p < 0,01 = **, p < 0,05 = *, ns, = not significant, Herbivory treatments are depicted by different symbols. Additional file 4: Table S1. Mean and ± SEM of volatile emission of frass and larvae, after removing them from the respective treatments. Values are expressed as nanograms emitted per gram of fresh weight per hour (ng/mg FW/h), GC-FID retention times for each compound are shown; unidentified compounds are labeled UN ID.
