# Supplementary material for: The timing of herbivore-induced volatile emission in black poplar (Populus nigra) and the influence of herbivore age and identity affect the value of individual volatiles as cues for herbivore enemies
Source: BMC Plant Biol. 2014 Nov 28;14:304. doi: 10.1186/s12870-014-0304-5 (PMC4262996; doi:10.1186/s12870-014-0304-5)
Supplement: Additional file 1: Figure S1. — Volatile emission pattern of thirteen further volatiles of Populus nigra foliage representing the major chemical classes released by young trees upon herbivory by fourth instar larvae of Lymantria dispar (gypsy moth) or from undamaged controls over a 4-day experiment. The graphs depict the rates of emission for individual compounds over the course of herbivory (initiated at the beginning of the experiment for herbivory treatment as well as after herbivore removal) during day and night in 6 h intervals. Means + SEM are given at the end of each measuring period. [file 12870_2014_304_MOESM1_ESM.pdf]

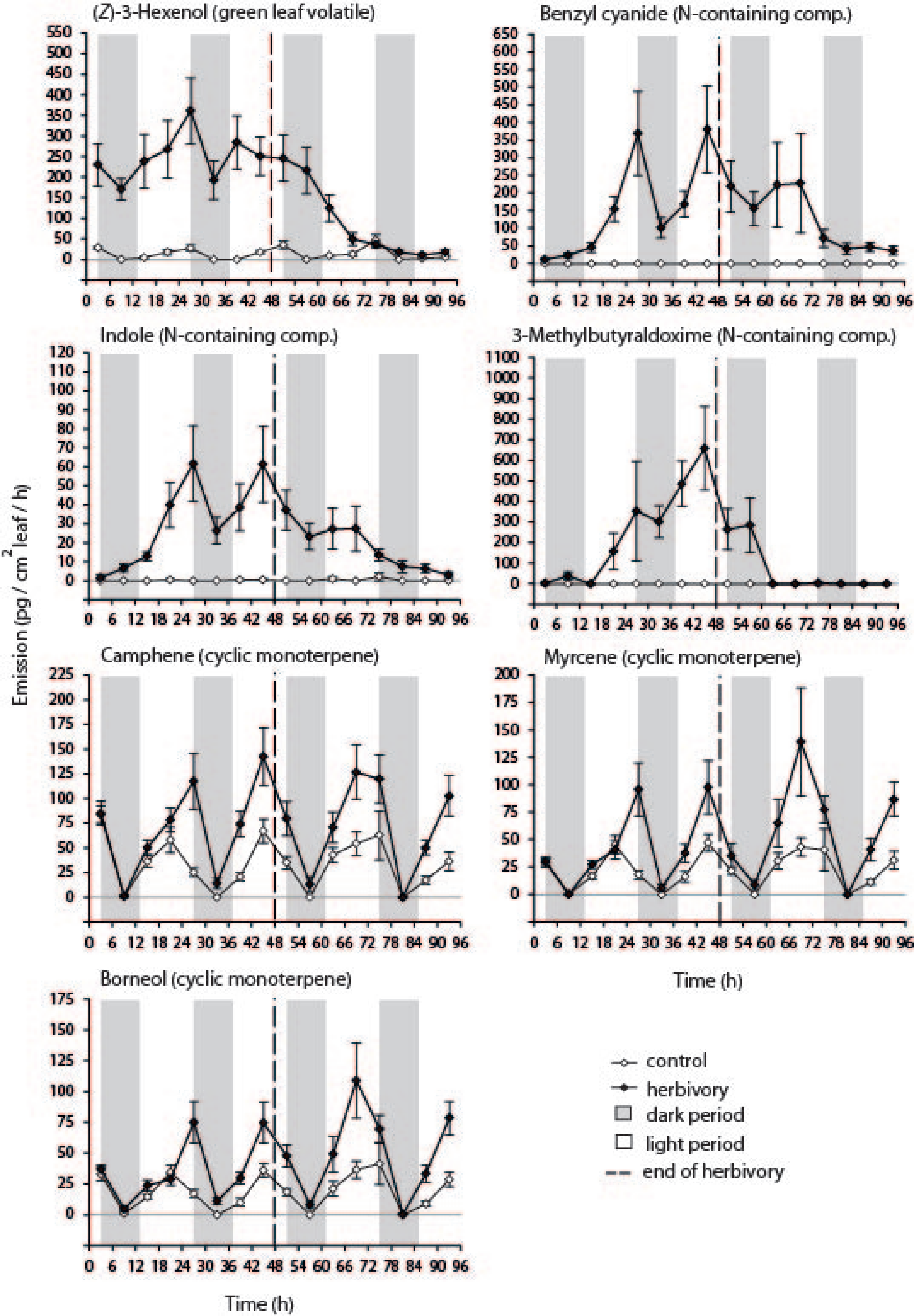

(Z)-3-Hexenol (green leaf volatile)
Benzyl cyanide (N-containing comp.)
Indole (N-containing comp.)
3-Methylbutyraldoxime (N-containing comp.)
Camphene (cyclic monoterpene)
Myrcene (cyclic monoterpene)
Borneol (cyclic monoterpene)
Emission (pg / cm2 leaf / h)
Time (h)
control
herbivory
dark period
light period
end of herbivory

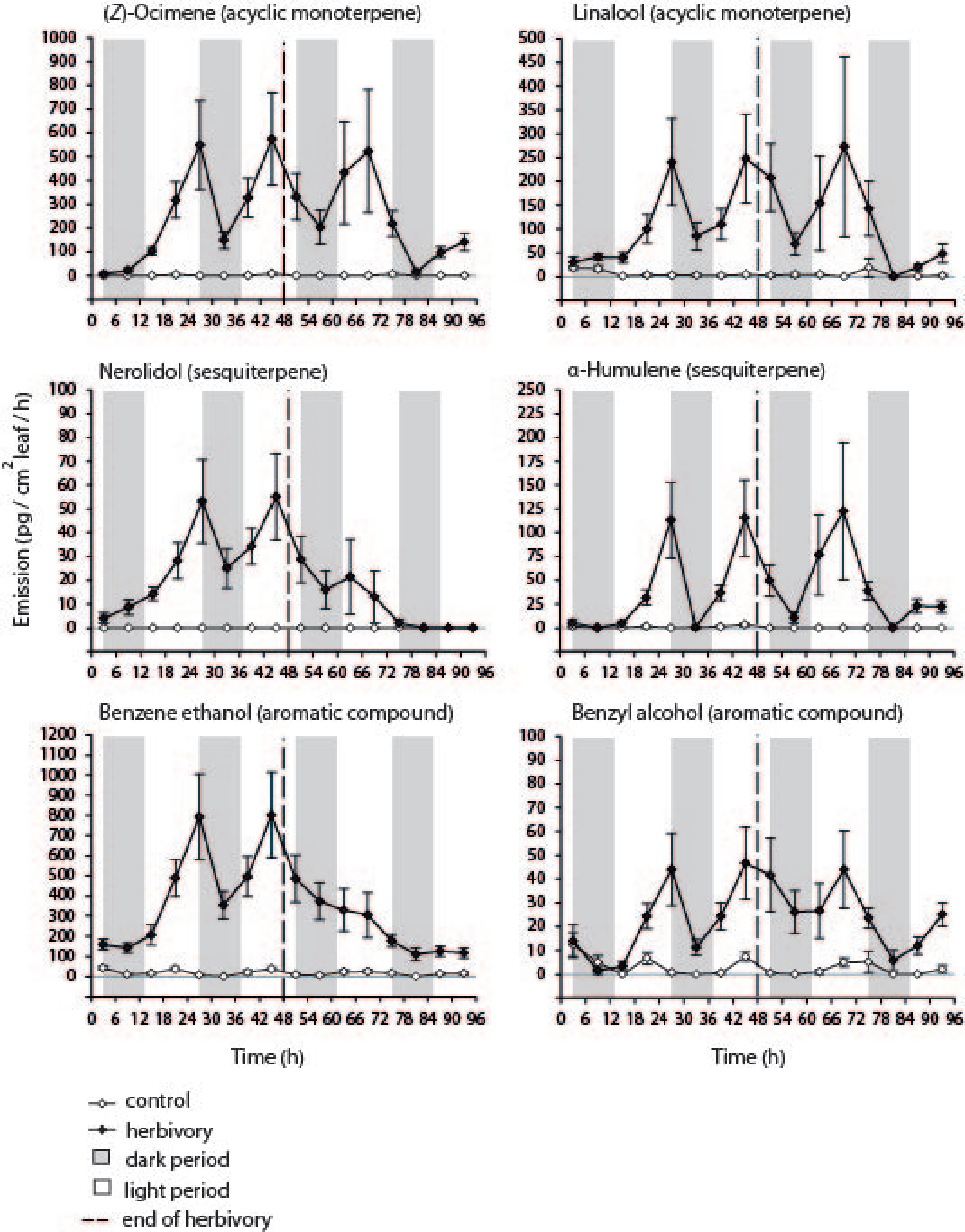

(Z)-Ocimene (acyclic monoterpene)
Linalool (acyclic monoterpene)
Nerolidol (sesquiterpene)
α-Humulene (sesquiterpene)
Benzene ethanol (aromatic compound)
Benzyl alcohol (aromatic compound)
Emission (pg / cm2 leaf / h)
Time (h)
Time (h)
control
herbivory
dark period
light period
end of herbivory
