# Supplementary material for: The timing of herbivore-induced volatile emission in black poplar (Populus nigra) and the influence of herbivore age and identity affect the value of individual volatiles as cues for herbivore enemies
Source: BMC Plant Biol. 2014 Nov 28;14:304. doi: 10.1186/s12870-014-0304-5 (PMC4262996; doi:10.1186/s12870-014-0304-5)
Supplement: Additional file 2: Figure S2. — Effect of herbivore identity and developmental stage on volatile emission of Populus nigra (for thirteen further volatile compounds), Four treatments include Lymantria dispar (2nd instar), L, dispar (5th instar), Laothoe populi (5th instar), and a mixture of L, dispar (5th instar) and L, populi (5th instar), Box-plots showing the same letter are not statistically significant from one another after a Tukey test performed on the fitted values after applying a GLS model, excluding the effect of the feeding intensity, P values are given in Table 1, Plots showing no letters indicate that there was no effect of the treatment on volatile emission. [file 12870_2014_304_MOESM2_ESM.pdf]

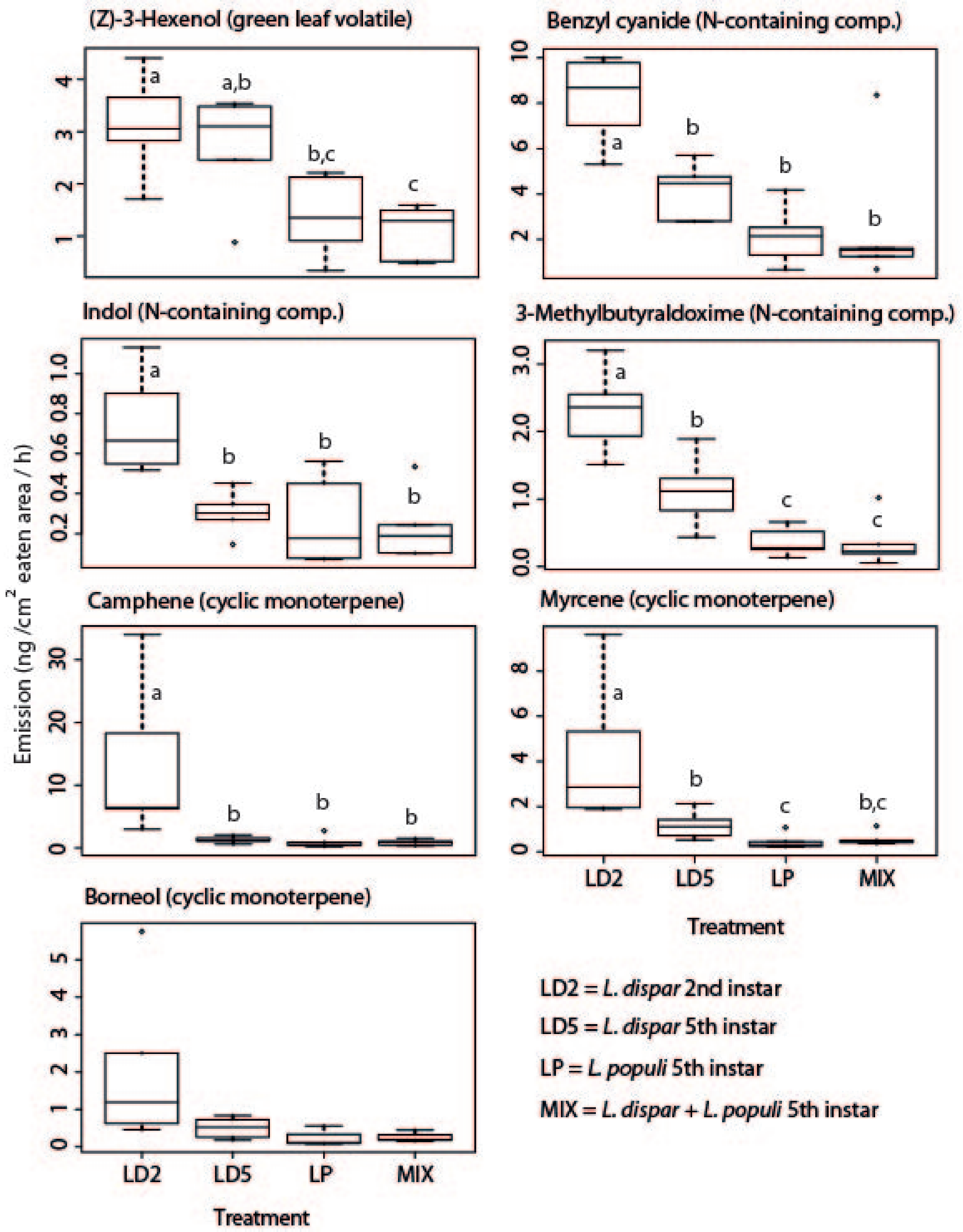
(Z)-3-Hexenol (green leaf volatile)
Benzyl cyanide (N-containing comp.)
Indol (N-containing comp.)
3-Methylbutyraldoxime (N-containing comp.)
Camphene (cyclic monoterpene)
Myrcene (cyclic monoterpene)
Borneol (cyclic monoterpene)
Emission (ng /cm2 eaten area / h)
LD2
LD5
LP
MIX
Treatment
LD2 = L. dispar 2nd instar
LD5 = L. dispar 5th instar
LP = L. populi 5th instar
MIX = L. dispar + L. populi 5th instar

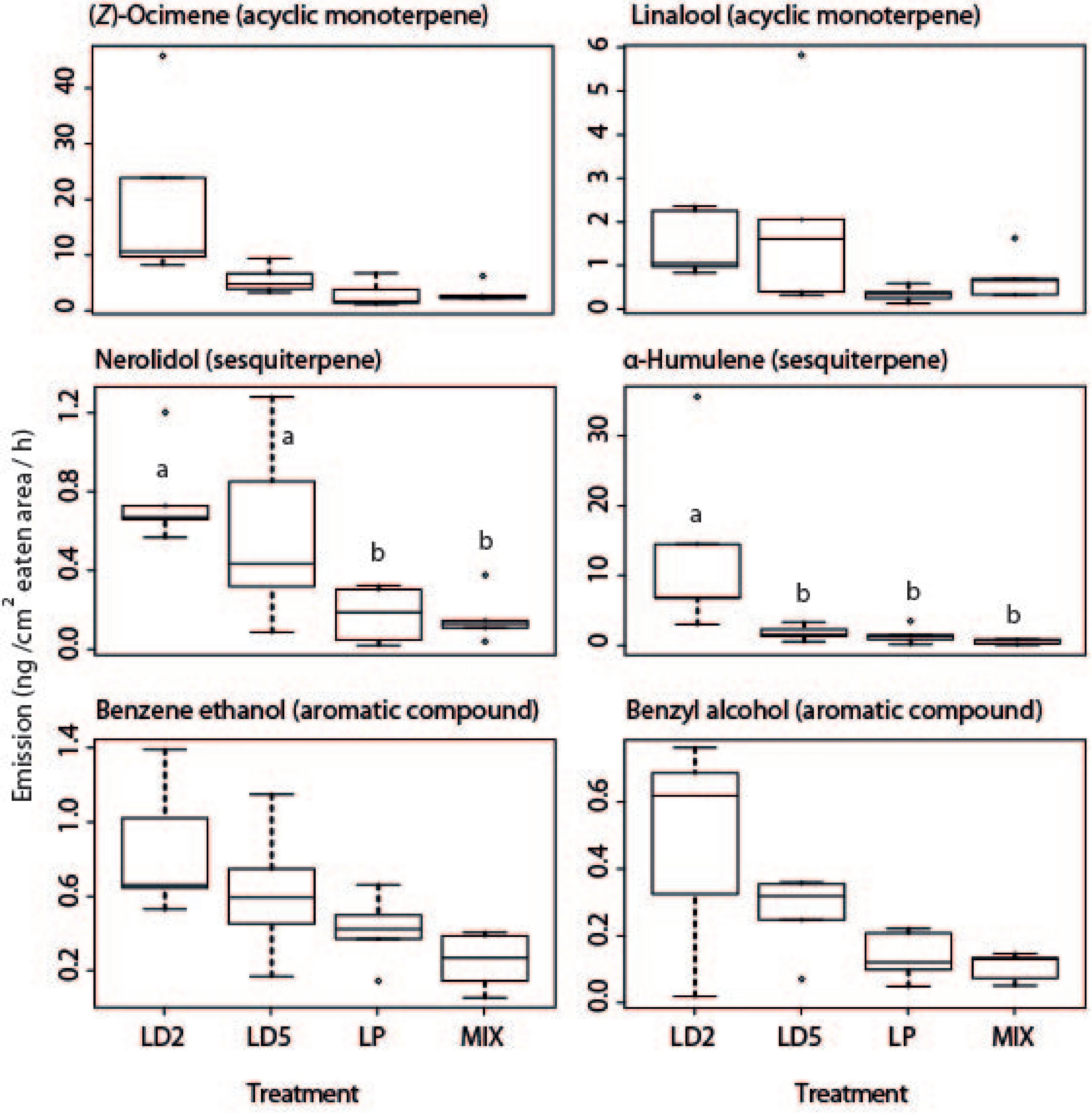

LD2 = *L. dispar* 2nd instar

LD5 = *L. dispar* 5th instar

LP = *L. populi* 5th instar

MIX = *L. dispar* + *L. populi* 5th instar
