# Supplementary material for: The timing of herbivore-induced volatile emission in black poplar (Populus nigra) and the influence of herbivore age and identity affect the value of individual volatiles as cues for herbivore enemies
Source: BMC Plant Biol. 2014 Nov 28;14:304. doi: 10.1186/s12870-014-0304-5 (PMC4262996; doi:10.1186/s12870-014-0304-5)
Supplement: Additional file 3: Figure S3. — Effect of feeding intensity during various herbivory treatments on volatile emission of Populus nigra compounds (for thirteen further volatile compounds), P values indicate significant differences after applying a GLS model (excluding the effect of herbivore identity), p < 0,001 = ***, p < 0,01 = **, p < 0,05 = *, ns, = not significant, Herbivory treatments are depicted by different symbols. [file 12870_2014_304_MOESM3_ESM.pdf]

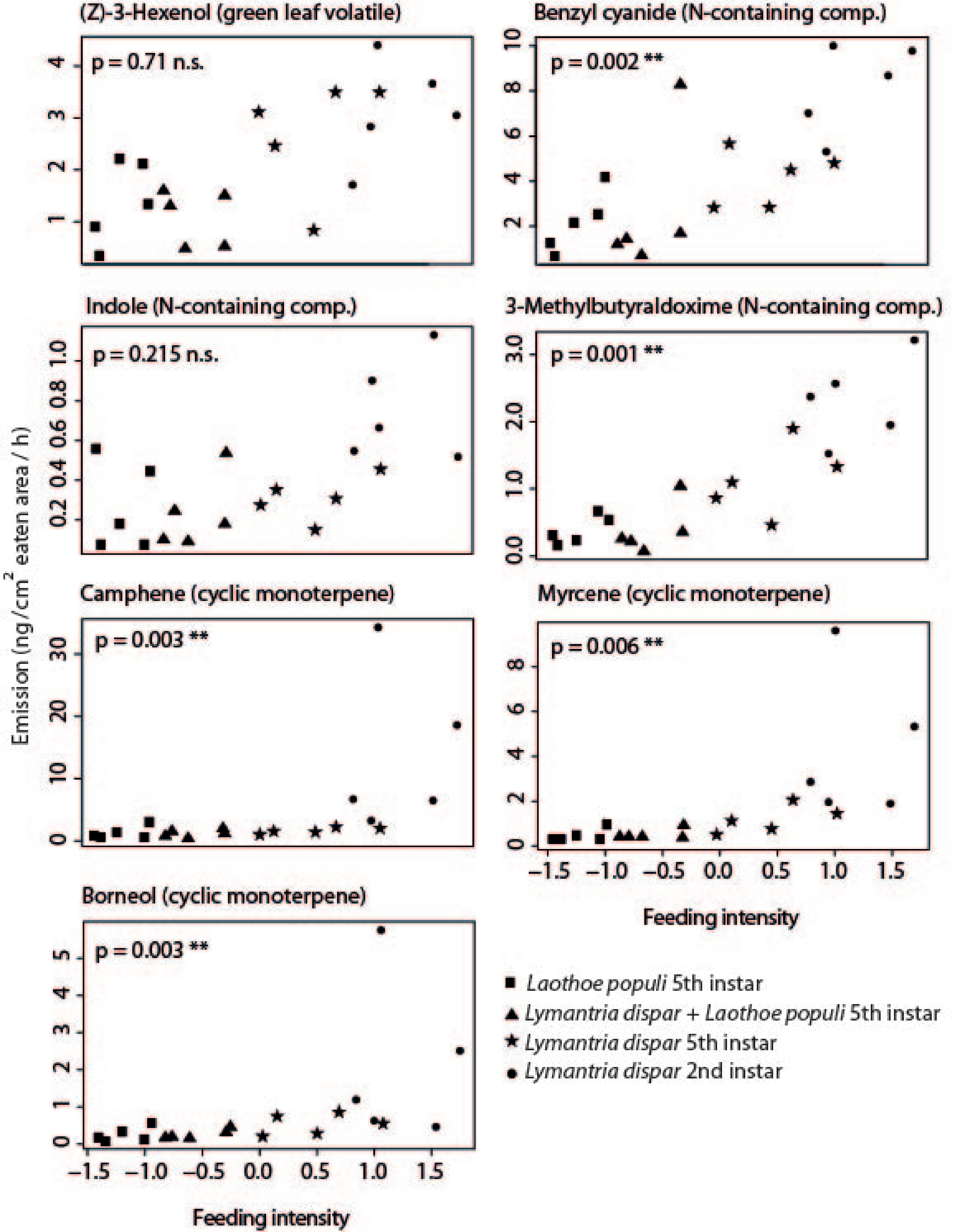

(Z)-3-Hexenol (green leaf volatile)
p = 0.71 n.s.
Benzyl cyanide (N-containing comp.)
p = 0.002 **
Indole (N-containing comp.)
p = 0.215 n.s.
3-Methylbutyraldoxime (N-containing comp.)
p = 0.001 **
Camphene (cyclic monoterpene)
p = 0.003 **
Myrcene (cyclic monoterpene)
p = 0.006 **
Borneol (cyclic monoterpene)
p = 0.003 **
Emission (ng/cm2 eaten area / h)
Feeding intensity
Laothoe populi 5th instar
Lymantria dispar + Laothoe populi 5th instar
Lymantria dispar 5th instar
Lymantria dispar 2nd instar

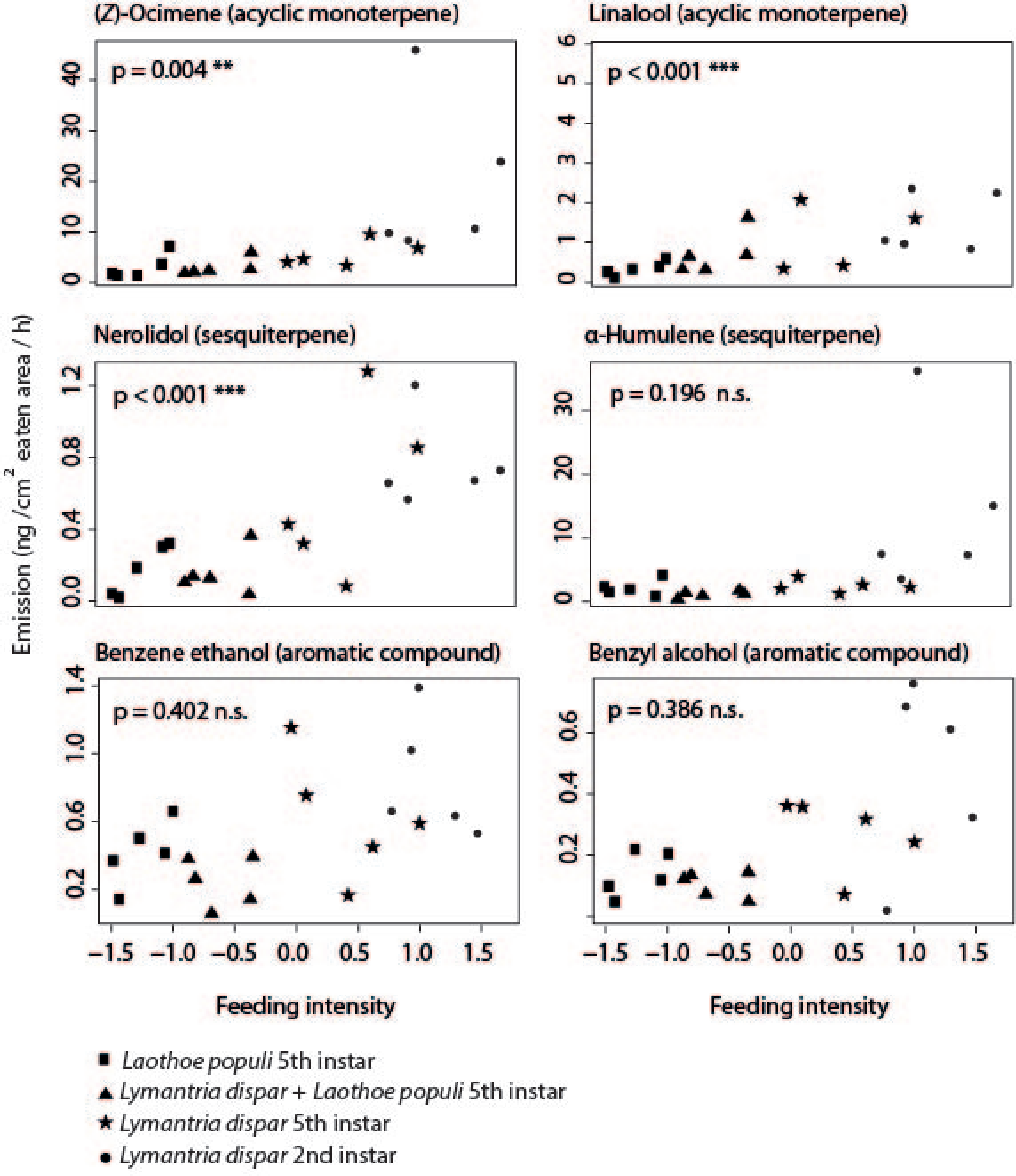
(Z)-Ocimene (acyclic monoterpene)
p = 0.004 **
Linalool (acyclic monoterpene)
p < 0.001 ***
Nerolidol (sesquiterpene)
p < 0.001 ***
α-Humulene (sesquiterpene)
p = 0.196 n.s.
Benzene ethanol (aromatic compound)
p = 0.402 n.s.
Benzyl alcohol (aromatic compound)
p = 0.386 n.s.
Emission (ng /cm2 eaten area / h)
Feeding intensity
Feeding intensity
Laothoe populi 5th instar
Lymantria dispar + Laothoe populi 5th instar
Lymantria dispar 5th instar
Lymantria dispar 2nd instar
