# Supplementary material for: The timing of herbivore-induced volatile emission in black poplar (Populus nigra) and the influence of herbivore age and identity affect the value of individual volatiles as cues for herbivore enemies
Source: BMC Plant Biol. 2014 Nov 28;14:304. doi: 10.1186/s12870-014-0304-5 (PMC4262996; doi:10.1186/s12870-014-0304-5)
Supplement: Additional file 4: Table S1. — Mean and ± SEM of volatile emission of frass and larvae, after removing them from the respective treatments. Values are expressed as nanograms emitted per gram of fresh weight per hour (ng/mg FW/h), GC-FID retention times for each compound are shown; unidentified compounds are labeled UN ID. [file 12870_2014_304_MOESM4_ESM.docx]

| **Emission (ng /g FW/h)** | | | | | | | | | |
| --- | --- | --- | --- | --- | --- | --- | --- | --- | --- |
|  |  | *L. populi* 5i | | *L. dispar* 5i | | *L. dispar* 2i | | Mixed herbivory | |
| **GC-FID** | **Compound** | MEAN | ±SEM | MEAN | ±SEM | MEAN | ±SEM | MEAN | ±SEM |
| 9.24 | UN ID | 3.99 | 0.29 | 7.66 | 1.8 | 57.51 | 11.07 | 2.66 | 0.21 |
| 9.32 | UN ID | 1.6 | 0.12 | 3.24 | 0.74 | 24.1 | 4.63 | 1.12 | 0.08 |
| 10.2 | UN ID | 4.79 | 0.3 | 8.71 | 2.14 | 67.88 | 13.91 | 3.09 | 0.26 |
| 10.34 | Farnesane | 1.98 | 0.15 | 3.46 | 0.83 | 28.07 | 6.16 | 1.26 | 0.13 |
| 15.31 | (*E*)-DMNT | 2.2 | 0.64 | 3.21 | 0.44 | 6 | 3.73 | 0.48 | 0.48 |
| 16.11 | 6-Methyl-5-hepten-2-one | 0.59 | 0.38 | 0.17 | 0.17 | 12.4 | 9.66 | 0.57 | 0.26 |
| 17.25 | (*Z*)-3-Hexenol | 2.79 | 1.3 | 12.23 | 3.3 | 16.63 | 7.38 | 3.16 | 0.62 |
| 17.5 | Nonanal | 1.86 | 0.48 | 4.68 | 1.71 | 34.2 | 7.19 | 0.83 | 0.12 |
| 17.72 | Hexanal | 3.4 | 0.94 | 6.18 | 2.36 | 4.91 | 3.02 | 2.39 | 0.43 |
| 18.7 | (*Z*)-Linalool oxide | 17.63 | 6.37 | 9.23 | 5.05 | 0 | 0 | 18.52 | 5.06 |
| 19.39 | (*E*)-Linalool oxide | 3.37 | 1.38 | 1.4 | 0.59 | 0 | 0 | 1.85 | 0.64 |
| 19.44 | (*Z*)-3-Hexenyl-2-methyl butanoate | 0.29 | 0.18 | 1.24 | 0.58 | 0 | 0 | 0 | 0 |
| 19.79 | 2-Ethyl-hexanol | 15.42 | 6.67 | 5.56 | 0.91 | 22.18 | 12.75 | 9.41 | 3.02 |
| 20.76 | Benzaldehyde | 1.3 | 0.5 | 4.91 | 2.18 | 8.07 | 4.18 | 2.28 | 0.91 |
| 21.18 | Linalool | 2.49 | 1.49 | 1.27 | 0.58 | 1.15 | 1.15 | 1.55 | 0.41 |
| 21.53 | 1,3 Butanediol | 0 | 0 | 0.99 | 0.82 | 0 | 0 | 0 | 0 |
| 22.68 | 2-Hydroxy ciclohexanone | 26.76 | 14.53 | 20.11 | 9.37 | 15.18 | 9.45 | 47.73 | 14.77 |
| 22.95 | β-Ciclocitral | 0.77 | 0.22 | 0.2 | 0.2 | 0 | 0 | 0.67 | 0.11 |
| 23.69 | Acetophenone | 5.48 | 2.08 | 0.45 | 0.31 | 0.72 | 0.72 | 4.23 | 1.47 |
| 24.37 | Salicyl aldehyde | 25.98 | 8.54 | 304.06 | 131.12 | 187.08 | 93.4 | 33.06 | 7.49 |
| 24.73 | Borneol | 25.66 | 9.77 | 1.21 | 0.95 | 0.69 | 0.69 | 12.47 | 6.46 |
| 25.54 | Epoxy linalool | 8.57 | 2.07 | 13.35 | 2.83 | 17.75 | 8.23 | 8.61 | 1.23 |
| 25.73 | (*E,E*)-α Farnesene | 1.08 | 0.29 | 4 | 0.92 | 10.64 | 5.31 | 0 | 0 |
| 26.43 | Methyl salicylate | 0 | 0 | 0.8 | 0.37 | 0 | 0 | 0 | 0 |
| 27.18 | UN ID | 1.38 | 0.97 | 1.71 | 0.89 | 0 | 0 | 1.69 | 0.66 |
| 27.56 | UN ID | 1.26 | 0.19 | 1.3 | 0.15 | 3.82 | 2.93 | 1.22 | 0.22 |
| 28.22 | UN ID | 1.56 | 1.05 | 0.72 | 0.48 | 2.63 | 2.63 | 0.49 | 0.23 |
| 28.33 | UN ID | 0.93 | 0.09 | 0.94 | 0.32 | 6.01 | 3.75 | 0.8 | 0.09 |
| 28.52 | Benzyl alcohol | 7.8 | 3.78 | 118.78 | 65.24 | 58.92 | 32.67 | 7.72 | 4.44 |
| 29.21 | Phenyl ethly alcohol | 3.1 | 0.82 | 15.39 | 5.65 | 8.56 | 5.45 | 1.52 | 0.64 |
| 29.52 | (*E*)-1,2-Ciclohexanediol | 2.47 | 0.77 | 7.96 | 4.88 | 24.92 | 17.25 | 3.86 | 2.4 |
| 30.3 | (*Z*)-1,2-Ciclohexanediol | 5.74 | 1.65 | 24.31 | 7.67 | 32.55 | 10.06 | 7.59 | 1.14 |
| 31.18 | Phenol | 1.15 | 0.61 | 9.45 | 5.2 | 16.76 | 16.76 | 4.93 | 2.46 |
| 32.17 | Isoeugenol | 2.24 | 1.4 | 0.36 | 0.36 | 0 | 0 | 0 | 0 |
| 32.8 | L-Proline | 1.17 | 0.49 | 16.28 | 5.19 | 83.5 | 21.76 | 3.36 | 0.86 |
| 34.11 | Eugenol | 49.66 | 16.65 | 31.83 | 5.65 | 60.4 | 12.59 | 21.13 | 5.65 |
